# Pathologic and molecular insights in nodal T-follicular helper cell lymphomas

**DOI:** 10.3389/fonc.2023.1105651

**Published:** 2023-01-30

**Authors:** Mario L. Marques-Piubelli, Catalina Amador, Francisco Vega

**Affiliations:** ^1^ Department of Translational Molecular Pathology, The University of Texas MD Anderson Cancer Center, Houston, TX, United States; ^2^ Division of Hematopathology, Department of Pathology and Laboratory Medicine, University of Miami, Miami, FL, United States; ^3^ Department of Hematopathology, The University of Texas MD Anderson Cancer Center, Houston, TX, United States

**Keywords:** peripheral T cell lymphomas, angioimmunoblastic T cell lymphoma, follicular T helper, next-generation sequencing, molecular and genetic profiling

## Abstract

T-follicular helper (TFH) cells are one of the T-cell subsets with a critical role in the regulation of germinal center (GC) reactions. TFH cells contribute to the positive selection of GC B-cells and promote plasma cell differentiation and antibody production. TFH cells express a unique phenotype characterized by *PD-1^hi^, ICOS^hi^, CD40L^hi^, CD95^hi^, CTLA^hi^, CCR7^lo^, and CXCR5^hi^*. Three main subtypes of nodal TFH lymphomas have been described: 1) angioimmunoblastic-type, 2) follicular-type, and 3) not otherwise specified (NOS). The diagnosis of these neoplasms can be challenging, and it is rendered based on a combination of clinical, laboratory, histopathologic, immunophenotypic, and molecular findings. The markers most frequently used to identify a TFH immunophenotype in paraffin-embedded tissue sections include PD-1, CXCL13, CXCR5, ICOS, BCL6, and CD10. These neoplasms feature a characteristic and similar, but not identical, mutational landscape with mutations in epigenetic modifiers (*TET2*, *DNMT3A*, *IDH2*), *RHOA*, and T-cell receptor signaling genes. Here, we briefly review the biology of TFH cells and present a summary of the current pathologic, molecular, and genetic features of nodal lymphomas. We want to highlight the importance of performing a consistent panel of TFH immunostains and mutational studies in TCLs to identify TFH lymphomas.

## Introduction

Nodal T-follicular helper (TFH) lymphomas represent a group of mature peripheral T-cell lymphomas (TCLs) with a gene expression profile and immunophenotype similar to that of nodal TFH cells (1, 2). This concept was initially described in angioimmunoblastic T-cell lymphoma (AITL) (3, 4) and more recently in a subset of peripheral T-cell lymphomas not otherwise specified (PTCL, NOS), which also has a gene expression profile, and immunophenotype suggestive of a TFH derivation (5, 6).

Here we present a brief summary of the biology of TFH cells and our current understanding of the clinicopathologic, molecular, and genetic features of nodal TFH lymphomas. Diagnostic considerations for this subgroup of lymphomas include distinguishing TFH lymphomas from other entities, particularly PTCL, NOS. For this, it is paramount to highlight the importance of performing a consistent set of at least 5 TFH immunomarkers and mutational studies in the work up of mature TCLs. Currently, we recommend using a five-marker panel that includes CD10, CXCL13, PD-1, ICOS and BCL6. This panel is used by other major academic centers in US and Europe. Per the WHO classification, the minimum criteria for assigning a TFH phenotype is 2 (but ideally 3 or more) markers in addition to CD4.

## T-Follicular helper cells

The differentiation of naïve CD4+ T-cells through the stimulation of antigen-presenting cells (APC) is an essential step for the maintenance of adaptive immunity homeostasis. The CD4+ helper T (Th) subtype is essential for the effector functions of the T-cells, and it can be divided into four major subsets of effector cells that produce distinct cytokines to help in the recruitment and activation of different cell types. These subsets include Th1 and Th2 cells (for type 1 and type 2 helper T-cells, respectively), Th17 cells (due to their IL-17 signature), and follicular helper T (TFH) cells. The latter is a fundamental component of the germinal center (GC) reaction and B-cell specialization (7, 8). Other T-cell subsets include T regulatory cells (Treg), Th9, and Th22 (9).

Phenotypically, TFH cells are characterized by the expression of BCL6 and CXC chemokine receptors type 5 (CXCR5), which allow them to reach the GCs (10, 11). TFH cells also express the inducible T-cell costimulator (ICOS), the programmed cell death protein (PD-1), and the signaling lymphocytic activation molecule (SLAM)-associated protein (SAP) that contribute to the crosstalk between T- and B-cells. Inherited mutations in the *ICOS* gene cause some antibody deficiencies (12).

TFH cells also provide crucial signaling for the normal ontogeny of B-cells. Engagements of MHC class II, CD40, and ICOS-ligand on GC B-cells with the TCR, CD40L, and ICOS on TFH cells may generate the production of IL-21, CD40L, and IL-4 and support the formation and maintenance of GCs (13). These signals allow antigen-specific B-cells to survive, proliferate, undergo affinity maturation, and, ultimately, differentiate into memory B-cells or long-lived plasma cells.

BCL6 is the master transcriptional regulator in the differentiation of TFH cells. BLIMP1 is a transcription factor that antagonizes BCL6 function and prevents TFH differentiation (14). BCL-6 also inhibits transcription factors important to other T-cell subsets, such as T-bet, Gata 3, and RORyt needed for Th1, Th2, and Th17 differentiation, respectively (15–17). BCL-6 also prompts the upregulation of CXCR5, a required step for the relocation of these cells toward the GCs (18).

TET2-mediated demethylation of DNA at specific regulatory regions is required to balance the differentiation of CD4+ T-cells towards Th1 and TFH lineages. In the absence of TET2, CD4+ T-cell differentiation is skewed toward the generation of highly functional TFH cells (19). Note that *TET2* loss-of-function mutations are frequently found in follicular helper T-cell lymphomas (see below).

Although TFH cells are needed for the GC reaction and therefore are located inside the GCs, they can also be found in other compartments outside the GCs. Circulating memory TFH cells appear to derive from GC TFH cells following downregulation of BCL6, ICOS, and PD-1 and upregulation of CCR7 (20–22). An activated subset of circulating TFH cells with expression of P-D1 and ICOS but with low expression of CCR7 has also been described (23). These circulating activated subsets of TFH cells are functional and associated with autoimmune processes (23, 24). T-follicular regulatory (Tfr) cells are another subset of TFH cells that share many characteristics of TFH cells, including the expression of BCL6, CXCR5, ICOS, and PD-1 but also express FOXP3, as conventional Tregs (25). Importantly, Tfr cells seem to have a suppressive function inside the GCs.

## Nodal T-Follicular helper cell lymphomas

In the revised 4th edition of the Classification of Hematolymphoid Neoplasms of the World Health Organization (WHO), three lymphoma entities with a TFH gene expression signature were included and designated as AITL, nodal PTCL with a TFH phenotype and follicular T-cell lymphoma (FTCL) and grouped under the provisional umbrella category of nodal lymphomas of TFH origin (**Table 1**) (26). In the updated classification (5^th^ Edition), these lymphomas are currently unified as nodal follicular helper T-cell lymphomas (TFH lymphoma) with three diseases, AITL-type, follicular-type, and not otherwise specified (NOS) (1). A similar approach and terminology have been adopted in the International Consensus Classification (ICC) (2). While the nomenclature is almost identical, there are minor differences, including the term nodal in the WHO, as implied in the ICC (**Table 1**) (1, 2). The immunomarkers most frequently used to establish a TFH immunophenotype in clinical practice include PD-1, CXCL13, CXCR5, ICOS, BCL6, and CD10.

**Table 1:** Different designations for nodal T cell lymphomas with a TFH immunophenotype according to the different classifications schemas.

WHO 2008	WHO 2017	ICC 2022	WHO 2022
	Nodal lymphomas of T-follicular helper cell origin	Follicular helper T-cell lymphoma	Nodal TFH cell lymphoma
Angioimmunoblastic T-cell lymphoma	*Angioimmunoblastic T-cell lymphoma*	Follicular helper T-cell lymphoma, angioimmunoblastic type	Nodal TFH lymphoma, angioimmunoblastic-type
Follicular variant of PTCL, NOS	Follicular T-cell lymphoma	Follicular helper T-cell lymphoma, follicular type	Nodal TFH lymphoma, follicular-type
Subset of PTCL, NOS including T-zone variant	Nodal peripheral T-cell lymphoma with TFH phenotype	Follicular helper T-cell lymphoma, NOS	Nodal TFH lymphoma, NOS

WHO, World Health Organization; ICC, International Consensus Classification; PTCL, peripheral T cell lymphoma, not otherwise specified; TFH, T-follicular helper.

### Nodal TFH cell lymphoma, angioimmunoblastic-type

For simplicity, we refer to this lymphoma as the historically used abbreviation AITL. AITL is the prototype of nodal TFH lymphomas, and it is one of the most common PTCL subtypes. There are some geographical variations in the frequency of AITL, with reported higher incidences in Europe than in the US; AITL in Europe represents about 35% of non-cutaneous T-cell lymphomas (TCLs) and ~16% in the US (27, 28). The diagnosis of AITL is based on a combination of clinical and laboratory findings, distinctive histopathologic features, and expression of TFH immunophenotype by the neoplastic cells. The neoplastic cells are characterized by the expression in variable rates of TFH markers such as CD10, Bcl-6, PD-1/CD279, ICOS, and CXCL13 (6, 26, 29). Other reported TFH markers that are not routinely used in clinical practice, include CXCR5, SAP, c-MAF, and CD200 (30–32).

#### Epidemiology and clinical features

AITL commonly affects middle-aged patients in the fifth to sixth decades, and there is a nearly equal incidence between genders and ethnicity. No racial predisposition is recognized. The most common clinical presentation is generalized lymphadenopathy (typically less than 3 cm), hepatosplenomegaly, constitutional symptoms, and skin rashes secondary to either neoplastic T-cell infiltration or as an autoimmune paraneoplastic manifestation (33). At diagnosis, most patients present with advanced stage and extranodal involvement. The most common sites of extranodal involvement are the spleen, bone marrow, skin, and lungs. Bone marrow involvement tends to occur early in the disease course, and its diagnosis can be extremely challenging (34). AITL is often associated with immune dysregulation, resulting in autoimmune complications and opportunistic infections (35, 36). The autoimmune and immunologic manifestations in TFH lymphomas are likely related to the functional and key role of TFH cells in B-cell activation, differentiation to plasma cells and B-cell recruitment. There are anecdotal reports of patients presenting with a smoldering course with waxing and waning lymphadenopathy (37, 38). While AITL is a systemic disease, rare cases of AITL and PTCL with TFH phenotype with localized disease and more indolent behavior have been reported, for example cases localized to the Waldeyer’s ring at presentation (39).

#### Morphologic features

**Figure 1:**
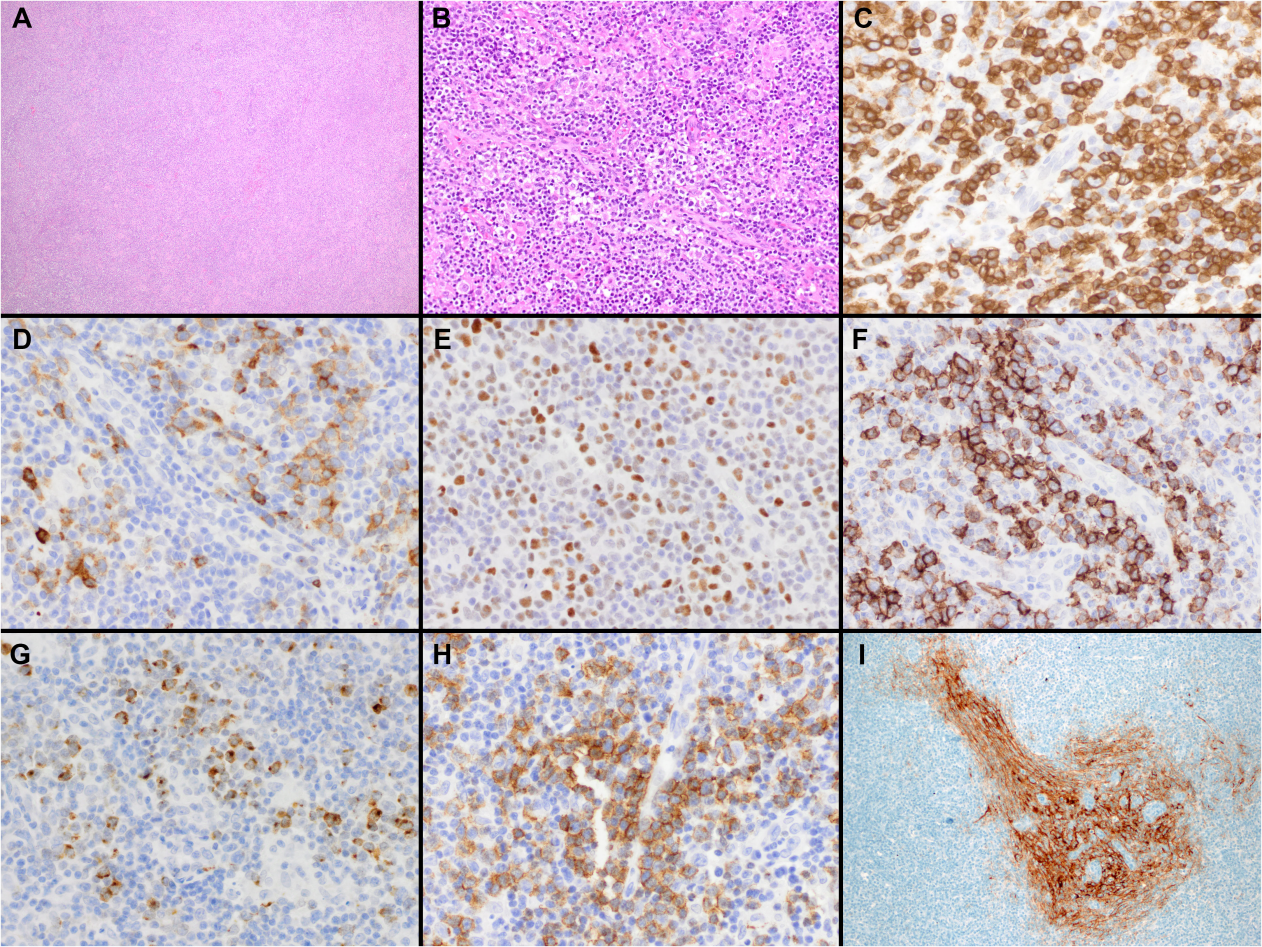
Histopathologic features of nodal T-follicular helper (TFH) cell lymphoma, angioimmunoblastic-type. **(A, B)** Hematoxylin & Eosin (H&E) shows that the neoplasm completely effaces the nodal architecture **(A)**; the neoplasm is diffuse and composed of a heterogeneous cell infiltrate associated with numerous high endothelial venules (HEVs), inflammatory cells, and clusters of small lymphocytes with clear cytoplasm **(B)** (2x and 40x); **(C)** CD3 shows that most of the cells in the infiltrate are T-cells. Some of the T-cells are intermediate in size with irregular nuclear contours (40x); **(D-H)** The tumor cells are positive for CD10 **(D)**, BCL6 **(E)**, ICOS **(F)**, CXCL13 **(G)** and PD-1 **(H)** supporting a TFH immunophenotype (all 40x). Note the clustering of the tumor cells around the HEVs. **(I)** CD21 highlights focally expanded follicular dendritic cell meshworks surrounding HEVs (20x).

Lymph nodes involved by AITL typically show partial or complete effacement with usually a diffuse or paracortical growth pattern and frequent perinodal extension but sparing of the subcortical sinuses (**Figure 1**). The neoplastic T-cells often constitute a minor part of a polymorphic inflammatory cellular infiltrate. The tumor cells are usually small- to medium-sized with mild nuclear atypia and clear cytoplasm. While they can be difficult to identify, they usually cluster around high endothelial venules (HEV) and are entrapped by the follicular dendritic cell (FDC) meshworks. Scattered or small groups of medium to large tumor cells with clear cytoplasm have been associated with *IDH2^R172^* mutations (40). Additional distinctive pathologic features include the proliferation of arborizing high endothelial venules (HEV) surrounded by expanded networks of follicular dendritic cells (FDCs), and an inflammatory background with plasma cells, eosinophils, histiocytes, and scattered B-cell immunoblasts. Plasma cells may be abundant, in rare cases obscuring the neoplastic T-cells, and are usually polyclonal but may be monoclonal in a few cases (41).

Three overlapping architectural patterns (types I, II, and III) have been described by Attygale and colls (29). Pattern III is the most frequent (~80%) and is characterized by a total effacement of the nodal architecture without follicles. Pattern II shows multiple regressive “burnout”/atretic germinal centers, and pattern I (reactive hyperplasia-like) is characterized by reactive hyperplastic germinal centers with the lymphoma located in the interfollicular regions and associated with minimal expansion of FDC. A tumor cell-rich pattern has been recognized and likely represents a histological progression in patients with previous AITL (42). This pattern is enriched with tumor cells and is associated with minimal inflammatory background and limited or no HEV proliferation simulating PTCL-NOS. Other histologic variants of AITL include epithelioid-rich AITL, plasma cell-rich AITL, and AITL with Hodgkin Reed-Sternberg (HRS)-like cells. The epithelioid-rich AITL is characterized by a high content of epithelioid cells simulating lymphoepitheloid lymphoma/Lennert lymphoma or a granulomatous reaction (43, 44).

Frequently, AITL contains a variable number of B immunoblasts, which can be EBV-positive or negative. In some cases, B-immunoblasts can be monoclonal and form confluent sheets of large B-cells that morphologically meet the diagnostic criteria of large B cell lymphoma (27, 42, 45, 46). More rarely, the associated clonal B-cell proliferations are characterized by small lymphocytes and/or plasma cells, which may resemble nodal or extranodal marginal zone lymphoma (47).

HRS-like B-cells are not unusual in AITL, which are more frequently EBV-positive (48). These HRS-like cells can be rosetted by neoplastic T-cells, express B-cell markers such as CD20, or occasionally have overlapping immunophenotype with classical Hodgkin lymphoma (CHL), posing a diagnostic difficulty (42, 48).

Several reactive lymph node conditions with predominant paracortical and/or interfollicular patterns may morphologically resemble TCL. These include viral-induced lymphadenopathies, drug reactions in patients receiving anticonvulsant therapy (most commonly diphenylhydantoin), antibiotics or antivirals, and vaccination-induced reactions, as well as other non-specific etiologies. Drug-induced hypersensitivity syndrome/drug reaction with eosinophilia and systemic symptoms (DRESS) syndrome (also called Dilantin-associated lymphadenopathy) is a drug-induced severe adverse reaction that can be associated with lymphadenopathy, mimicking AITL (49).

#### Immunophenotypic features

The neoplastic T-cells are positive for alpha/beta TCR and CD4 and typically express pan-T cell markers, including CD2, CD3, and CD5. The aberrant loss or downregulation of one or more T-cell markers is frequently observed (50). The markers used to identify a TFH immunophenotype in paraffin-embedded tissue sections include PD-1, CXCL13, CXCR5, ICOS, BCL6, and CD10. PD-1 and ICOS are more sensitive than CXCL13 or CD10; conversely, CXCL13 and CD10 are more specific (51, 52). Partial expression of CD30 by the tumor cells is not unusual, and aberrant expression of CD20 by the lymphoma cells has also been reported (53). High expression of CD20 has been reported to be associated with a better overall survival (54–56). The use of follicular dendritic cell markers (e.g., CD21, CD23, or CD35) is helpful to assess for perivascular expansion of FDCs. CD23 has been recommended when staining for CD21 fails to show perivenular expansion of the FDCs (42). AITL with *IDH2* mutations has stronger expression of CD10 and CXCL13. An antibody against *IDH2^R172K^* is available and can detect most cases with this mutation (57).

Flow cytometry frequently reveals decreased or absent expression of surface CD3 (58, 59). Also, the detection of a T-cell population by flow cytometry coexpressing CD4/CD10 or CD4/PD-1 (bright) on lymph nodes, bone marrow, or peripheral blood samples may help to support the diagnosis (60).

There have been occasional reports of AITL with cytotoxic phenotypes (61) and the possibility of a florid CD8+ cytotoxic cell proliferation obscuring a neoplastic TFH cell population has also been reported (62).

#### Molecular features

##### Mutational profile

AITL frequently shows a distinctive mutational profile with mutations involving *RHOA^G17V^* (50-70%), *TET2* (40-80%), *IDH2^R172^* (20-45%) and *DNMT3A* (20-30%) (**Table 2**) (5, 55, 56).

**Table 2:** Mutations associated with nodal follicular helper T cell lymphomas.

Genes	Frequency			
	AITL	nTFH-NOS	nTFH-FL	
GTPase				
*RHOA^G17V^*	50-70	25-50	60	• G17V specific to AITL/PTCL-TFH
				• Not associated with prognosis
Epigenetic regulators				
*TET2*	40-80	50-75	75	• Found in other neoplasms (myeloid)
*DNMT3A*	20-30	7-18	25	• *TET2* co-occur w/ *DNMT3* and *IDH2* mutations is specific to TFH lymphomas
*IDH2^R172^*	20-45	0	0	• *IDH2* mutations mostly restricted to AITL • Presence of clear cells • More pronounced TFH signature • Strong CD10 and CXCL13 expression • Chr 5 and 21 gains • More aberrant genome than IDH2 negative cases • Clinical trial: enasidenib
TCR signaling pathway				
*PLCγ*	8-14	6.25	N/A	• Not specific (PTCL-NOS)
*CD28*	10-12	0	N/A	• Worse prognosis in AILT

N/A, Not available.

*RHOA* is a small GTPase involved in T-cell migration, polarization, and antigen recognition by cycling between GDP-bound (inactive) and GTP-bound (active states). The c.50G>T (p.Gly17Val) is the most frequent missense mutation of *RHOA* in AITL (56, 63). Mutations in *RHOA* ^G17V^ act as dominant negatives interfering with the signaling initiated by wild-type *RHOA* (64) and seem to be a secondary event contributing to the differentiation toward TFH phenotype (65). These mutations frequently co-occur with mutations in epigenetic regulators, especially *TET2* mutations. Mutated *RHOA* acquires a novel function, binding and phosphorylating VAV1 protein (66). *RHOA^G17V^* mutation seems to increase TCR signaling through enhanced VAV1 resulting in stronger TCR signaling and preferential commitment to TFH rather than non-TFH lineage through enhancing ICOS signaling (67).

*IDH2* is a metabolic mitochondrial enzyme involved in the generation of 2-oxoglutarate (2-HG). Mutant *IDH2* forms have a neomorphic enzymatic activity leading instead to the generation of 2-hydroxyglutarate (2-HG), an oncometabolite that antagonizes the activity of α-KG-dependent dioxygenases (histone demethylases and the TET family of 5mC hydroxylases) (68, 69). They are described as a secondary event and might refine the differentiation of the premalignant clones towards a TFH signature (70). *IDH2^R172^* mutations are associated with cases with a more pronounced TFH signature, strong CD10 and CXCL13 expression, gains of chromosomes 5 and 21, and more aberrant genome than the cases without *IDH2^R172^* (40, 71). The more aberrant genome seen in cases with *IDH2^R172^* mutations is likely due to the inhibitory effect of 2-HG oncometabolite on DNA repair enzymes (72). AITL with wild-type *IDH2* show significant enrichment of PI3K-AKT activation pathways and have focal losses of negative regulators (phosphatases) of the PI3K-AKT pathway (71).

*TET2*, also known as ten-eleven translocation 2 (TET2), encodes a 2-oxoglutarate/Fe2+-dependent oxygease that participates in the epigenetic control of gene expression by catalyzing the oxidation of DNA 5-methylcytosine to 5-hydroxymethylcytosine (73, 74). Its loss-of function is an initial event in the neoplastic transformation and is associated with a worse outcome (75). *DNMT3A* encodes a DNA methyltransferase that controls cytosine methylation. Loss-of-function mutations in *DNMT3A* are also considered an initial event in the transformation process and frequently co-occur with *TET2* mutations (69).

Other recurrent mutations frequently seen in AITL include TCR signaling genes, such as *VAV1, PLCG1, CD28*, and *FYN* (76). Those mutations, except for specific mutation and fusions of CD28, are not specific to AITL or TFH lymphomas. Mutations in the *CD28* gene appear to show implications in outcomes since CD28-mutated AITL patients have inferior survival compared to patients with wild-type CD28 (77). Alterations in *RHOA* and *VAV1* are mutually exclusive.

##### Relationship with clonal hematopoiesis

*TET2* and *DNMT3A* mutations are not specific to AITL. However, different from other neoplasms these two mutations frequently co-occur in AITL. *TET2* and *DNMT3a* mutations are the most frequent mutations in clonal hematopoiesis (CH). Many studies have now established that up to 80% of patients with TFH lymphomas carry the same *TET2* and/or *DNMT3A* mutations identified in the T-cells and the myeloid cells (78–80). Furthermore, *TET2* and *DNMT3A* mutations are not restricted to T-cells and myeloid cells but can also be identified in the admixed mature B-cells in the lymph nodes. On the contrary, *RHOA* and *IDH2* mutations appear to be confined to the neoplastic T-cells and represent the “second” hit that contribute to the T-cell lymphomagenesis (65, 79, 81).

The background of clonal hematopoiesis of indetermined potential (CHIP) appears to be the source of myeloid neoplasm seen in TCL with a TFH phenotype, particularly after cytotoxic therapy (78, 79, 82). The occurrence of AML and other myeloid neoplasms after the diagnosis of AITL is significantly high (79). From a practical perspective, it is important to remember that in a patient being followed for AITL the presence of CHIP mutations does not necessary imply the presence of residual lymphoma.

##### Gene expression profile

In AITL, the expression profiling signatures are enriched in genes typically expressed by TFH cells (55, 56, 71). This demonstration of molecular similarities between AITL cells and TFH cells at a genome-wide level established the cellular “derivation” of AITL from TFH cells, initially suspected based on the expression of single TFH markers in AITL cells (3, 4, 6).The molecular profile of AITL is also dominated by a strong microenvironment imprint, including overexpression of B-cell and FDC-related genes, chemokines/chemokine receptors, and genes related to the extracellular matrix and vascular biology (52, 83, 84). Gene expression studies identified oncogenic pathways, including the NF-κB pathway, IL-6 signaling, and the TGFβ pathway enriched in AITL compared to other PTCLs (3, 4, 6, 55), but the genetic etiology of these activated pathways has not been completely elucidated.

Recently, Amador and colls have developed a digital gene expression classifier using specific signatures in nodal T-cell lymphomas and included a specific signature for nodal AITL (54). This assay can be used in paraffin-embedded tissue sections and thus can easily be translated to routine clinical practice to complement our conventional pathology approaches to better classify nodal TCLs.

### Nodal TFH cell lymphoma, follicular type

For simplicity, we refer to this lymphoma as the historically used abbreviation FTCL. These are nodal TCLs with a nodular growth pattern that show significant histologic, immunophenotypic, transcriptomic, and genetic overlap with AITL (5). Isolated FTCL is very rare and usually occurs in association with AITL. Some patients at disease presentation have typical histologic and clinical features of AITL but, at relapse, show features of FTCL or vice versa (42, 85). Therefore it has been proposed that these two entities may constitute different morphologic representations of the same biological process (1, 2).

#### Epidemiology and clinical features

The median age of onset is 60-65 years with a slight male predominance (2). The clinical syndrome resembles AITL and other TFH lymphomas and is characterized by advanced-stage disease, generalized lymphadenopathy, splenomegaly, B symptoms, skin rash, and occasionally immune manifestations (85). Cases with localized disease have also been reported (86).

#### Morphologic features

**Figure 2:**
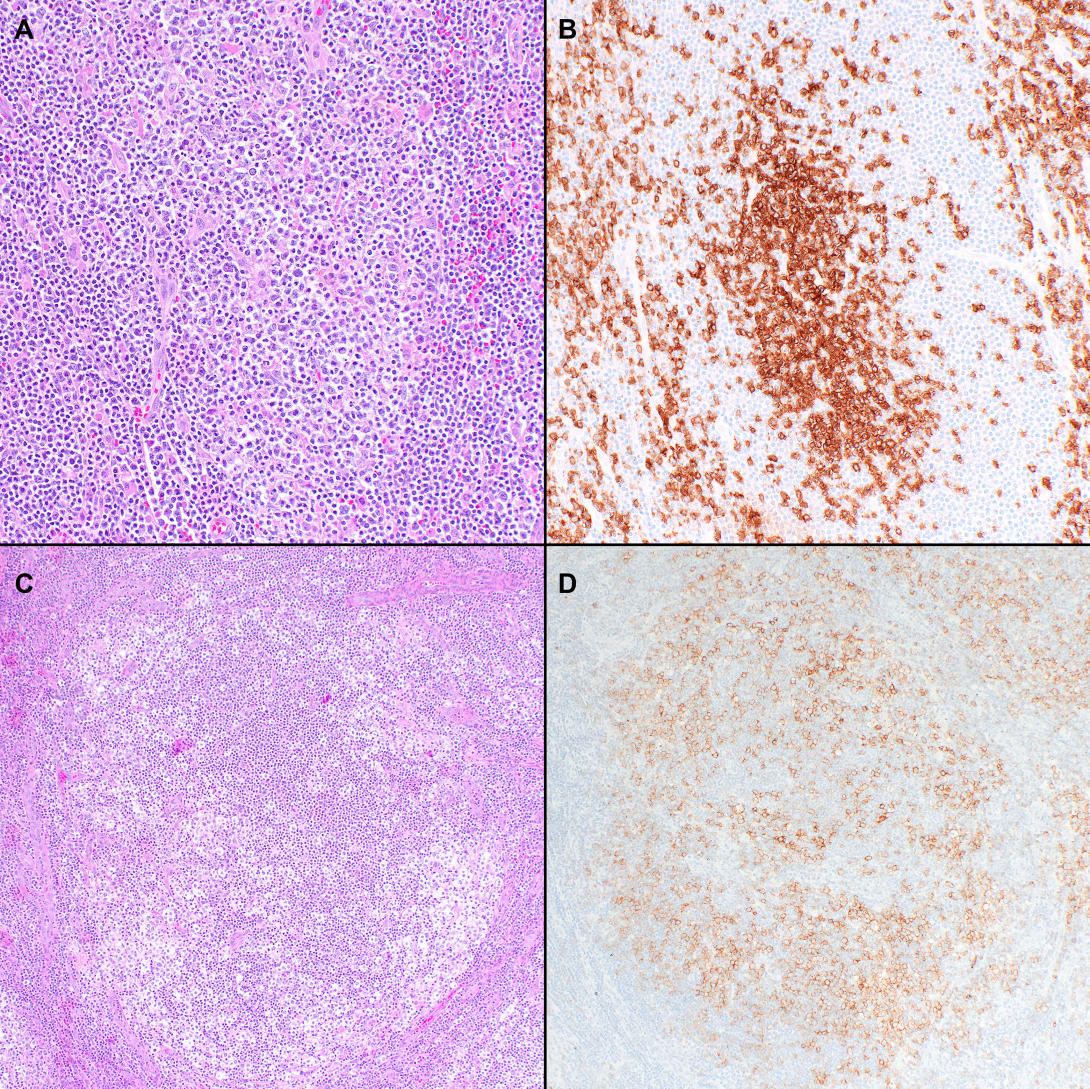
Histopathologic features of nodal T-follicular helper (TFH) cell lymphoma, follicular-type. **(A)** This case shows a predominantly follicular growth pattern, simulating follicular lymphoma, with the neoplastic follicles composed of small to medium-sized atypical lymphocytes admixed with scattered large cells (20x); **(B)** PD-1 shows that the tumor cells form solid clusters inside the nodules with residual B-cells pushed to the periphery of the follicles (not shown) (20x); **(C)** This other case shows that the neoplastic nodules have features of progressive transformation of germinal centers (PTGC). The nodules display a ‘moth-eaten’ appearance with aggregates of neoplastic T-cells surrounded by small B-cells (20x); **(D)** The neoplastic cells are positive for PD-1 in addition to other TFH markers (not shown) (20x).

Two patterns have been described. In the classic pathology description, FTCL has a follicular growth pattern mimicking follicular lymphoma (FL), where the follicles are populated by aberrant T-cells that express TFH markers (**Figure 2**) (85). Residual B-cells can be seen and are usually pushed to the periphery of the follicles by the neoplastic T-cells. Hodgkin and Reed-Sternberg (HRS)-like cells are also frequently noted. Alternatively, it can also mimic progressive transformation of germinal centers (PTGC). In this pattern, the nodules display a ‘moth-eaten’ appearance with aggregates of the neoplastic T-cells surrounded by small B-cells (85). Mixed FL-like and PTGC-like patterns can be seen. Focal paracortical hyperplasia is present with a polymorphic infiltrate of eosinophils and plasma cells and hyperplastic high endothelial venules.

#### Immunophenotypic features

The neoplastic T-cells have a TFH phenotype and typically express pan-T cell markers, including CD2, CD3, and CD5, although aberrant loss or downregulation of CD7 can be seen. By flow cytometry, dim expression of surface CD3 is frequently observed. CD4 is positive in most cases, with few instances double negative for CD4 and CD8 (85). The HRS-like cells are of B-cell lineage, positive for CD30 and positive for CD15 and EBER in some cases. This phenomenon raises concern for classic Hodgkin lymphoma. Neoplastic rosetting by T-cells around the HRS-like cells is seen in virtually all cases of FTCL, and a retained network of FDCs is seen underlying the neoplastic follicles. FTCL usually lacks the other typical features of AITL, including the characteristic expanded FDC networks surrounding HEVs (85).

#### Molecular features

The few cases included in gene expression profiling studies have shown that FTCL clusters closer to AITL than PTCL-NOS (5). Similarly, the mutational profile of FTCL seems to be similar to other nodal TFH lymphomas with mutations in *TET2, DNMT3A*, and *RHOA^G17V^*, but not in *IDH2* which are usually restricted to AITL (5). FTCL can harbor a characteristic t(5;9)(q33;q22) resulting in an *ITK-SYK* fusion in approximately 40% of cases (87). This fusion acts as a constitutively active SYK tyrosine kinase and drives lymphomagenesis by triggering antigen-independent activation of TCR signaling. Translocations involving *FER* and *FES* have been recently described, including *ITK-FER* and *RLTPR-FES*, that result in the activation of the STAT3 signaling (88).

### Nodal TFH cell lymphoma, not otherwise specified

Nodal TFH lymphoma, NOS includes those TCLs with TFH phenotype, confirmed by the expression of CD4 and at least 2 TFH markers that lack the morphologic features of AITL and FTCL. This group includes cases previously categorized as PTCL-NOS that have shown significant molecular and genetic overlap with other TFH lymphomas (5, 56, 71). It is possible that TFH lymphoma, NOS, represents more than a single entity as currently defined.

#### Epidemiology and clinical features

The frequency of this neoplasm is unknown. However, it is our personal experience that up to 30% of the PTCL-NOS are reclassified as nodal TFH, NOS when a panel of TFH immunomarkers is analyzed. The patients usually present with disseminated lymphadenopathy associated, which can be associated with autoimmune manifestations (5).

#### Morphologic features

**Figure 3:**
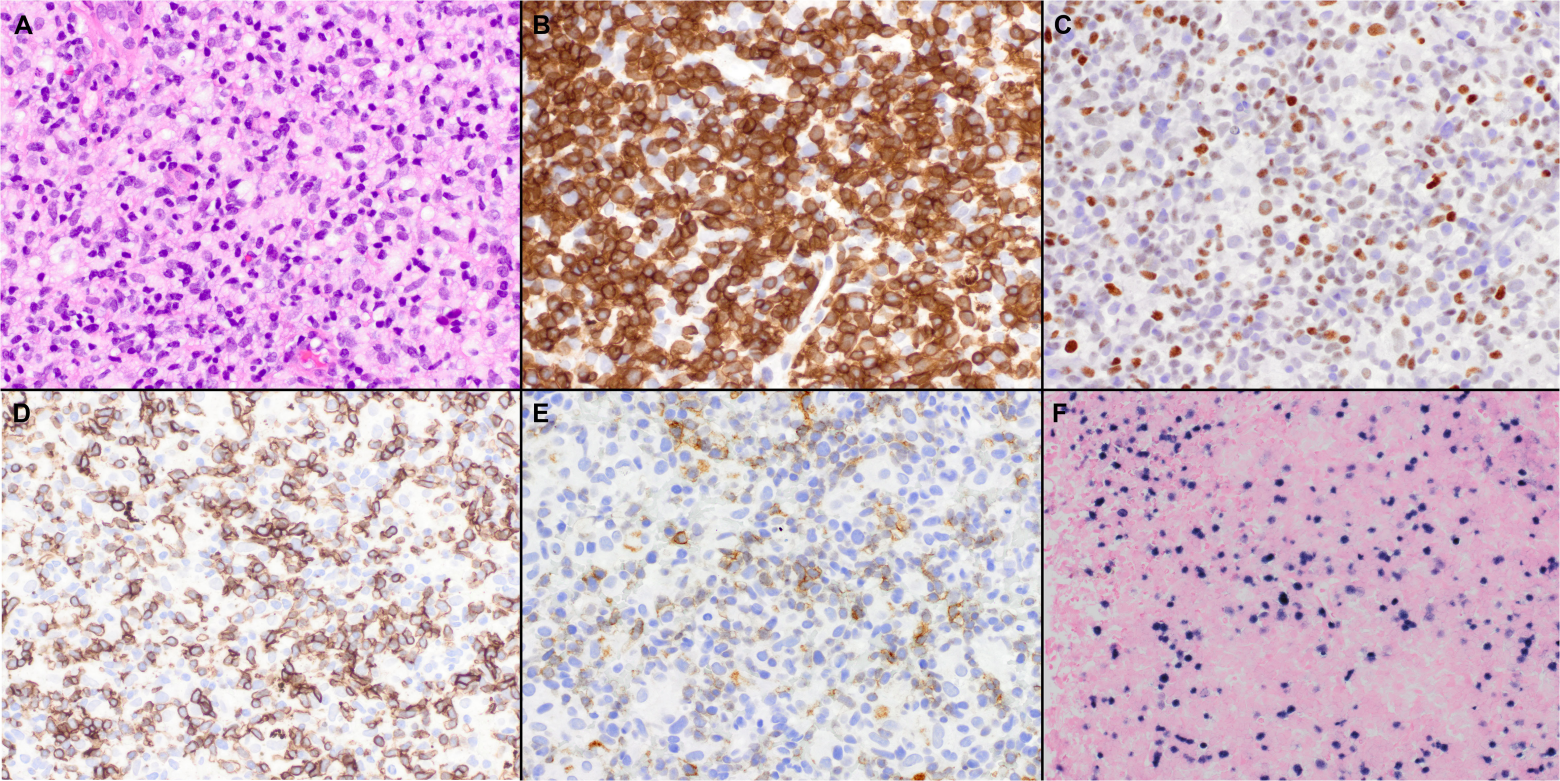
Histopathologic features of nodal TFH cell lymphoma, not otherwise specified (NOS). **(A)** In the case shown, the neoplasm is diffuse and composed predominantly of intermediate atypical lymphoid cells (40x); Features of AITL were not seen. **(B)** The tumor cells are positive for CD3 (40x); **(C-E)** BCL6 **(C)**, ICOS **(D)**, and PD-1 **(E)** are also variably positive in the neoplastic cells (40x); **(F)** Epstein-Barr virus-encoded small RNAs (EBER) shows positivity in scattered cells (20x).

The overall morphologic spectrum of these tumors has not been completely elucidated. Most cases are characterized by a diffuse tumor-cell-rich infiltrate of variably-sized lymphoid cells without the typical HEC and FDC proliferations (**Figure 3**) (1, 2). Additionally, cases previously described as lymphoepithelioid lymphomas with TFH marker expression are currently included in this group (89). A systematic evaluation of these cases shows that they can frequently have one or two AITL-like features not commonly seen in PTCL-NOS cases (51). There are some overlapping features between nodal TFH lymphoma, NOS and the tumor cell-rich pattern of AITL. However, it has been mentioned that the tumor cell-rich pattern of AITL still maintains the focal perivenular FDC expansions (1).

#### Immunophenotypic features

By definition, the tumor cells are positive for CD4 and express at least 2 TFH makers. Some cases show focal positivity for TFH markers and positivity in a small subset of the tumor cells, being difficult to classify. Currently, there is no standard percentage of TFH positivity and the intensity of expression in tumor cells needed to establish a reproducible diagnosis of TFH lymphoma, NOS over PTCL, NOS.

#### Molecular features

Similarly to FTCL, gene expression studies show that TFH lymphoma, NOS clusters closer to AITL than PTCL-NOS (5, 76). The mutational profile of TFH lymphoma, NOS, seems similar to AITL mutations in *TET2, DNMT3A*, and *RHOA^G17V^* (5, 56, 76). *IDH2^R172^* mutation is characteristic of AITL, although it can rarely be seen in TFH lymphoma, NOS (90). *RHOA^G17V^* mutations were identified in 60% of the cases (76). *TET2* mutations seem slightly more frequent in TFH lymphoma, NOS (5). Mutations in genes of the TCR signaling pathway (including CD28) can be seen in a subset of cases (76, 90). It has been reported that the presence of TCR-signaling-related mutations correlated with early disease progression (76).

## Conclusions

TFH lymphomas are a group of mature peripheral T-cell lymphomas with distinctive clinicopathologic and molecular features. Although AITL is well-characterized and has unique morphologic features that facilitate its diagnosis, the other subtypes, mainly the TFH lymphoma, NOS, is one of exclusion. Additional studies are required to better understand and delineate this category of nodal TFH lymphomas.

Since the diagnosis of nodal TFH lymphomas is very challenging, especially in small core needle biopsies, the diagnostic evaluation of TCLs requires the integration of clinicopathologic features together with a complete panel of TFH markers. We envision that incorporating mutational studies and gene expression profiling in the near future into the routine diagnostic armamentarium will facilitate the diagnosis of TFH lymphomas.

## Author contributions

All the authors performed writing, reviewing, and revisions of the manuscript. All authors contributed to the article and approved the submitted version.
